# Advances in Endoscopic Visualization of Barrett's Esophagus: The Role of Confocal Laser Endomicroscopy

**DOI:** 10.1155/2012/493961

**Published:** 2012-03-14

**Authors:** Helga Bertani, Flavia Pigò, Emanuele Dabizzi, Marzio Frazzoni, Vincenzo Giorgio Mirante, Mauro Manno, Raffaele Manta, Rita Conigliaro

**Affiliations:** Endoscopy Unit, Nuovo Ospedale Civile S. Agostino Estense, Via Giardini 1355, Baggiovara, 41126 Modena, Italy

## Abstract

Many endoscopic imaging modalities have been developed and introduced into clinical practice to enhance the diagnostic capabilities of upper endoscopy. In the past, detection of dysplasia and carcinoma of esophagus had been dependent on biopsies taken during standard white-light endoscopy (WLE). Recently high-resolution (HR) endoscopy enables us to visualize esophageal mucosa but resolution for glandular structures and cells is still low. Probe-based confocal laser endomicroscopy (pCLE) is a new promising diagnostic technique by which details of glandular and vascular structures of mucosal layer can be observed. However, the clinical utility of this new diagnostic tool has not yet been fully explored in a clinical setting. In this paper we will highlight this new technique for detection of esophageal dysplasia and carcinoma from a clinical practice perspective.

## 1. Introduction

Esophageal adenocarcinoma has the fastest growing incidence rate (300% increase over the past 4 decades) [1–3], and the risk of patients with Barrett's Esophagus (BE) of developing adenocarcinoma is 30–120 times greater. BE is a change in the distal esophageal epithelium of any length that can be recognized as columnar-type mucosa at endoscopy and is confirmed to have intestinal metaplasia by biopsy of the tubular esophagus [4]. The currently accepted paradigm correlates the risk of progression to the grade of dysplasia as there is evidence that progression occurs in an orderly fashion from no dysplasia to low-grade dysplasia (LGD) to high-grade dysplasia (HGD) followed by early esophageal adenocarcinoma [5]. In addition esophageal adenocarcinoma has a poor survival rate (<5% at five years) due to a late diagnosis, to an early vascular and lymphatic infiltration and to low vascularization of neoplastic tissue, which leads to a low response to chemotherapy of the tumor [4]. 

Since BE is considered the most important risk factor for the development of esophageal adenocarcinoma, assuming that the detection of mucosal dysplasia is critically important in patients with Barrett's oesophagus, because early diagnosis can prevent the progression to invasive carcinoma, international societies of gastrointestinal diseases suggest keeping patients in endoscopy surveillance program [5–7].

However, surveillance endoscopy has several limitations because dysplastic changes occurring in BE are not easily identifiable by standard endoscopy.

In the last decades, many technologic advances have been done in the field of endoscopic imaging, through HR endoscopy to magnification endoscopy to virtual chromoendoscopy, in order to achieve a better visualisation of mucosal layer and to distinguish neoplastic versus nonneoplastic tissue. But even if good, new techniques are not strong enough to replace biopsies. Consequently, the current standard of endoscopic practice is to take multiple biopsies because there are no features on standard or HR endoscopy that distinguish Barrett's glandular metaplasia, dysplasia, or early-stage neoplasia. However the accuracy of standard white light endoscopy (WLE) and random biopsies is low and may fail to detect neoplastic lesions [7]. Moreover biopsies obtained using this technique are prone to sampling error, and interobserver agreement is low even between advanced operators and even among expert pathologists [8, 9]. This results often in: (1) delay in reaching the final diagnosis and the decision of the correct and best treatment, (2) increased costs in pathology procedures, and (3) repeated procedures. In addition, sensitivity and specificity of histology are variable for difficulty to reach specimen adequacy. Moreover the presence of inflammation or ulcers could alter the mucosal architecture and give some false negative/positive results to pathology examination [10–14]. A multiple biopsies protocol could also interfere with next therapeutic steps; endoscopic mucosal resection (EMR) or endoscopic submucosal dissection (ESD) could be more difficult without adequate “lifting sign” due to scar tissue after repeated biopsies.

Nevertheless another important limitation of histology is that it is a postmortem analysis and it is not able to give us information about in vivo processes (blood flow).

Confocal laser endomicroscopy (CLE), a recent advance of endoluminal imaging, allows an in vivo visualization of mucosal layer with a detailed visualization of tissue and subcellular structures with magnification up to 1000 times. Since 2004 many papers, about the potential role of this new technique, have been published, and many studies have been introduced to validate this technique.

CLE has the potential to anticipate the final diagnosis (neoplastic versus nonneoplastic) and potentially to guide next therapeutic steps in clinical practice without the delay of a pathology response. Moreover it offers the possibility to study mucosal layer to a micron resolution giving us an “optical biopsy”. However, other technologies, such as narrow band imaging (NBI), autofluorescence imaging (AFI), and chromoendoscopy, are needed as “red-flag” techniques to initially detect and localize suspicious areas.

One of the potentially future applications of pCLE is about a role in in vivo study of physiologic and pathologic processes, like inflammation or angiogenesis in healthy or neoplastic tissue [15].

Currently two devices are available and approved by European Medicines Agency (EMA) and Food and Drug Administration (FDA) to perform CLE: one system is inserted in tip of the scope (eCLE, Pentax Corporation, Tokyo, Japan) and one, a probe-based system, is a separate device from the endoscope but able to be introduced in the working channel of any standard endoscope (pCLE, Cellvizio, Mauna KeaTech, Paris, France).

**eCLE**

In this system, the miniaturized confocal scanner has been integrated into the distal tip of a new endoscope. A blue laser light source delivers an excitation wavelength of 488 nm and light emissions detected at >505 nm. Successive points within the tissue are scanned in a raster pattern along *X*-axis and *Y*-axis to construct serial en face optical section of 475 × 475 *μ*m at user-controlled variable imaging depth. The optical slice thickness is 7 *μ*m with a lateral resolution of 0.7 *μ*m. Images on the screen approximate a 1000-fold magnification of the tissue in vivo [16]. The advantage of this system is that the working channel of the scope is free, and it can be used for target biopsies or for combined enhancement techniques such as chromoendoscopy. The limit of this system is that the calibre of the scope is bigger than a standard 11.8 mm upper scope and is stiff. Moreover the lens of the scope is not combined with HR software and virtual chromoendoscopy or other system (ISCAN).

**pCLE**

this system can be used through the working channel of any standard endoscope (colonoscope, gastroscope, cholangioscope, bronchoscope, and ureteroscop, etc). The advantage of this probe-based CLE is the versatility of the system and the possibility to combine it with other advanced “red flag” imaging modalities such as virtual chromoendoscopy or magnification. Scanning rates is 12 images/sec. The limits of this system pCLE are the slightly low power resolution compared to eCLE (1 mm versus 0.7 mm) and a small field of view (240–600 mm). So pCLE system is not well suited to surveying large areas of tissue such as long segments of BE and should ideally be combined with a red-flag technique for classification of tissue in a site already detected by enhanced endoscopy. However Mauna Kea has developed a postacquisition specifically-developed software (“mosaicing”) to paste images together and to obtain images similar to histology specimen.

## 2. Classification

An important issue for a new imaging technique is the standardization of terminology and classification of images. The first published classification about confocal imaging was the “Mainz classification” based on eCLE [17]. However, due to several technical differences between pCLE and e-CLE, in 2011 a new classification based on pCLE has been published after a consensus of pCLE experts held in Miami in 2009 [18].

**Figure 1:**
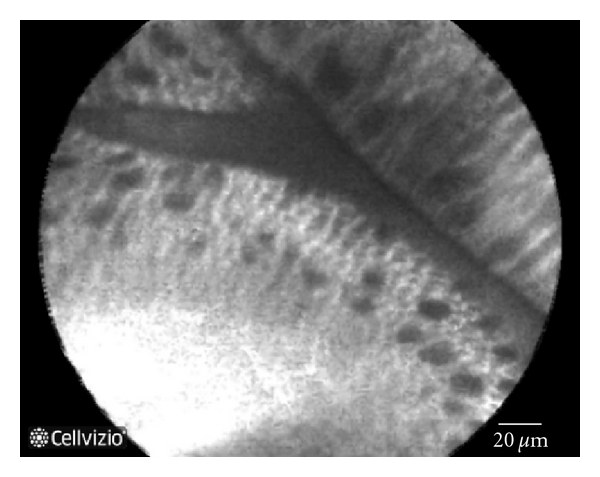
Normal Barrett's mucosa at pCLE: Villiform structure with goblet cell and regular epithelial lining.

**Figure 2:**
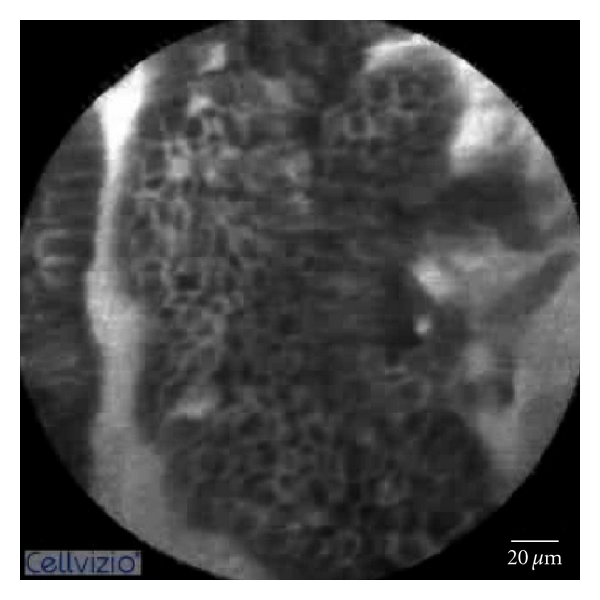
Dysplastic Barrett's mucosa. Villiform structure with dark and irregularly thickened epithelial lining.

pCLE shows detailed images including squamous epithelium, glandular architecture, crypts, columnar cells, goblet cells, and capillaries with red blood cells. In patients with a normal squamous epithelium pCLE shows flat cells without crypts, or villi, bright vessels within capillary loops. In case of BE diagnosis, pCLE shows the villiform architecture, columnar cells, and the presence of goblet cells (Figure 1). If the BE is complicated with dysplasia pCLE shows villiform structures with dark, irregularly thickened epithelial borders, dilated irregular vessels. In case of adenocarcinoma disorganized/loss of villiform structure and crypts dark columnar cells and dilated irregular vessels are found (Figure 2) [18].

## 3. Barrett's Esophagus Surveillance

One of the first and major clinical applications of pCLE is BE surveillance or, with a therapeutic approach, the definition of lesion's margin before EMR or ESD.

First published data (noncontrolled trials) showed that pCLE was able to detect intraepithelial neoplasia with a sensitivity of 75% and specificity of 89–91% [19]. In the same paper, ranking study population for disease-risk, in the low-risk group population, pCLE has a NPV nearly 98.8% suggesting the possibility to avoid random biopsies. However, a false-positive rate for suspected dysplasia of 64.3% using endomicroscopy was commented, and the authors replied that the study was not designed to calculate the performance characteristics (sensitivity, specificity, accuracy) [20]. In a pilot study by Pohl et al. the author evaluated the preliminary accuracy of pCLE for high-grade dysplasia (HGD) and adenocarcinoma in BE patients. They evaluated 296 sites in a 38-patient group. The overall accuracy of pCLE was 88% to 93% with a sensitivity of 75% to 80% and specificity of 89% to 94%, a PPV of 44.4%, and a NPV of 98.8% [21]. Kiesslich et al. using the eCLE system achieved an accuracy of 96.8% in BE metaplasia diagnosis and an accuracy of 97.4% in neoplasia diagnosis [17].

Another study by Bajbouj et al. [22] did not confirm these data, and the authors explained the differences with previous results with the low frequency of neoplasia detected in the study and secondly to strict adherence with diagnostic criteria for neoplasia in their data. The prevalence of neoplasia was lower than in the published data using the CLE system or other studies evaluating different imaging modalities, which have described prevalence of HG dysplasia or early cancers ranging between 24% and 59%. The authors also face the problem of overinterpretation in those studies, and the possibility of false positive pCLE findings increases [20].

Dunbar et al. [19] also demonstrated an increased yield of neoplasia compared to 4-quadrant biopsy protocol. Although the sensitivity of pCLE found in this study was lower than found in some previous studies, this study used a real-time prediction of histology by a great number of endoscopists from different Endoscopic Academic centers, which used different criteria in pCLE imaging interpretation, which may have led to variable results.

Recently Sharma et al. published the first international multicenter prospective randomized controlled trial [23]. The authors demonstrated significantly improved sensitivity in detection of HGD/EC using pCLE. The study involved 5 international centers, and 122 patients were enrolled. Of the enrolled patients, 21 were excluded from the analysis. A twofold increased sensitivity in detections of HGD/EC for HD-WLE compared to HD-WLE or pCLE (34.2% to 68.3%, resp.) was shown. This translated into 41 additional locations with HGD/EC being identified when pCLE was used in conjunction with HD-WLE compared with HD-WLE alone [23].

## 4. Barrett's Esophagus Treatment

Another recent application of confocal endomicroscopy is a role in therapeutic endoscopic procedures. The management of BE with neoplasia (HGD/“early cancer”) ranges from surgery to endoscopic treatment and is evolving to include multiple endoscopic modalities in order to increase the rates of esophagus-sparing therapies. Recently, the trend is not only to treat the dysplastic BE but also to eradicate the remaining at-risk Barrett's epithelium to prevent metachronous and synchronous lesions. Endoscopic therapies include both tissue-acquiring (EMR, ESD) and non-tissue-acquiring therapies (RFA and photodynamic therapy). The results of the above-mentioned therapies are very different in the published series; EMR demonstrated a long-term experiences and good results in terms of radicality at the expense of high complications rates such as perforations and strictures [24, 25]. RFA and cryotherapy offer promising results, though the latter still lacks significant follow-up time [26]. But in this great number of emerging therapies, the appropriate selection of an endoscopic modality for the treatment of a lesion in clinical practice is based upon endoscopist's experience and new technologies' availability. pCLE can play a role in (a) localization of lesions and prediction of pathology, (b) in targeting biopsies and resections in surveillance and treatment, and (c) in the choice of therapy to use. Konda et al. recently published a case series [27] showing a possible role of pCLE in therapeutic endoscopy. This case series illustrates a range of cases in which CLE was used during the procedure and offered the chance of providing real-time information during endoscopic treatment or follow-up. Thus, the endoscopist may have endomicroscopic information, and in the future pCLE could be used to tailor BE management strategy between surveillance, biopsies, ablation, or resection-based strategies.

## 5. Interobserver Agreement and Learning Curve

Issues to consider before introducing a new technique in clinical practice include the learning curve for the endoscopist and interobserver agreement.

Wallace et al. [28] evaluated the accuracy and inter-observer agreement of 9 international endoscopists in pCLE in patients with BE-associated dysplasia. The overall accuracy of pCLE for the diagnosis of HGD was 90.5%, sensitivity 88%, and specificity 94%. If endoscopist had previous experience in endomicroscopy imaging interpretation, the overall accuracy was of 97%, sensitivity 94%, and specificity of 100%. The overall interobserver agreement was 0.72; 95% (CI 0.57–0.85). A matter of contention is the need of a real-time interpretation that may be different from reviewing sequences after image acquisition.

## 6. Conclusions

The promising results recently published are potentially changing the role of endoscopist into an “endomicroscopist”. However, for any emerging technique, more data are needed to confirm the results in clinical setting, to evaluate the possible increased diagnostic accuracy of HR endoscopy and the possibility to reduce the missing rate of dysplastic BE in surveillance program. Another issue will be the applicability of a new technique outside research program and the costs, if applied, in surveillance program.
